# Inspiring the future generation of oncologists: a UK-wide study of medical students’ views towards oncology

**DOI:** 10.1186/s12909-021-02506-0

**Published:** 2021-02-02

**Authors:** Kathrine S. Rallis, Anna Maria Wozniak, Sara Hui, Marios Nicolaides, Neha Shah, Beena Subba, Apostolos Papalois, Michail Sideris

**Affiliations:** 1 Barts and The London School of Medicine and Dentistry, Queen Mary University of London, London, UK; 2 Newham University Hospital, Barts Health NHS Trust, London, UK; 3 North Middlesex University Hospital NHS Trust, London, UK; 4 Experimental Educational and Research Centre ELPEN, Athens, Greece; 5 Women’s Health Research Unit, Queen Mary University of London, Yvonne Carter Building, 58 Turner Street, London, E1 2AB UK

**Keywords:** Medical students, Oncologists, Undergraduate medical education, Curriculum, Surveys and questionnaires

## Abstract

**Background:**

One in 2 people born in the UK after 1960 are expected to require oncology input in their lifetime. However, only 36% of UK medical schools provide dedicated oncology placements and teaching indicating a discordance between public health impact and training. We designed a UK-wide survey to capture medical students’ views on current oncology teaching and the potential role of a national undergraduate oncology symposium as an educational, networking and motivational tool.

**Methods:**

We undertook a national cross-sectional survey of UK medical students’ views in oncology and satisfaction with teaching using pre-designed questionnaires. We also distributed a dedicated survey (pre and post-conference) to compare medical students’ motivation towards a career in oncology after attending the national symposium. This study was prospectively approved by QMUL Ethics Committee (Reference number QMREC2348). Statistical analysis included univariate inferential tests on SPSS and GraphPad software.

**Results:**

The national survey was completed by 166 students representing 22 UK medical schools. Students reported limited interest, knowledge and exposure to oncology, lack of confidence in skills, and teaching dissatisfaction. Oncology was perceived as a challenging specialty (mean 4.5/5 ± 0.7), yet most students estimate receiving only 1–2 weeks of dedicated oncology teaching. The national symposium generated a statically significant increase in students’ interest, knowledge, and confidence in skills surrounding oncology, improving students’ perceived ability to cope with the emotional challenges in this field.

**Conclusion:**

Students’ views towards oncology alongside their teaching dissatisfaction underpin the need to revisit and strive to improve current undergraduate oncology curricula. Increasing medical student oncology exposure by proposing outcome-based guidelines and adopting a standardised undergraduate oncology curriculum should be the foremost priority in inspiring future oncologists to ensure excellent cancer patient care.

**Supplementary Information:**

The online version contains supplementary material available at 10.1186/s12909-021-02506-0.

## Highlights of this study

- To our knowledge, this is the first national cross-sectional survey of UK medical students’ views in oncology.
- Data collection from the national survey represented several institutions throughout the UK providing valuable insight into students’ views, capturing various different angles.
- Our described intervention (the National Undergraduate Oncology Conference) was successful in increasing medical students’ interest, knowledge, and confidence in skills towards oncology.
- Future work should focus on generating outcome-based guidelines to reform undergraduate oncology teaching curricula within medical schools in order to inspire future oncologists and meet increasing societal cancer care demands.

## Background

Cancer is the second leading cause of death worldwide pertaining an imminent global health threat. Latest estimates suggest a 1 in 2 lifetime risk of developing cancer in those born in the UK after 1960 [1]. According to The Edinburgh Declaration of the World Conference on Medical Education, medical education should “reflect the needs of the defined society in which it is situated” [2]. All practitioners will inevitably treat cancer patients as incidences rise [3, 4]. Thus, exposure to oncology is fundamental regardless of specialisation. Most general health of cancer patients is managed by non-cancer-specialists including newly-qualified doctors and primary care physicians with limited or no postgraduate oncology training [4–6].

Despite rising global cancer burden and an increasing global demand for oncologists to join the workforce [7, 8], several studies report ongoing underrepresentation and lack of consistent undergraduate oncology teaching [9–14] with few universities offering dedicated oncology teaching blocks [15–17]. As far as we know, UK medical schools rarely distinguish between medical, clinical/radiation, and surgical oncology teaching within undergraduate curricula as these are often discussed collectively in the context of specific diseases. Oncologists are concerned about medical students’ cancer-related skills and knowledge, especially considering its fragmented teaching across systems leading to factual omissions [14, 18, 19]. Although several extra-curriculum teaching programmes, including conferences and extended week courses, run across several European countries and overseas [20–23], there is still an evident need to reform undergraduate oncology teaching curricula to meet societal and clinical practice needs [24–26].

Therefore, the aims of this study were to (1) survey national medical student views on oncology including their knowledge, confidence in skills, and satisfaction with oncology teaching in undergraduate medical education and (2) evaluate the impact of a national undergraduate oncology conference on students’ views on oncology.

## Methods

Our evaluation included a national cross-sectional mixed survey seeking Likert scale, multiple choice, binary yes/no, and free text responses. We also performed a comparative targeted-group pre and post-conference quasi-experimental survey seeking similar responses.

### Participants

#### National survey

Medical students attending any of the 41 recognised UK medical schools were invited to complete an electronic questionnaire disseminated during March–May 2020, advertised in two cycles. The survey was distributed via the Medical Schools Council (MSC) to UK medical school staff who then disseminated the survey to student cohorts. The survey was also promoted via student association channels, including newsletter and social media.

#### Pre/post-conference questionnaires

Medical students attending the National Undergraduate Oncology Conference, London, 7th March, 2020, were invited to complete two questionnaires each distributed via email using three reminder rounds for each, 1 week before and after the conference respectively. The pre-conference questionnaire was also available electronically and in paper format at conference registration.

### Intervention

#### Questionnaires

Questionnaires were designed according to a literature search of oncology questionnaires aimed at medical students. Discussions between research team members contributed to the final questionnaire components and design. The national survey assessed students' views towards oncology in five domains including: career interest, emotional attitudes, knowledge or exposure, confidence in skills, and teaching satisfaction. The national survey and pre-conference questionnaire (Appendix 1 and 2 respectively) contained identical questions; the post-conference questionnaire (Appendix 3) contained an extra section dedicated to feedback. Excluding questions pertaining to participant demographics, each questionnaire compromised 34 questions sub-divided into views on oncology, and views on oncology teaching, including 32 questions on a 5-point Likert scale, one multiple choice, and one free text response.

#### Conference 

The National Undergraduate Oncology Conference was designed as a supplementary educational tool by lecture-based format. Smaller group-based workshops encouraged networking and allowed for exchange of ideas between students, but also with clinicians and patients. A CV-building workshop served as a signpost for career avenues within hospital-based and academic oncology.

### Comparisons

We assessed responses across geographical location, medical school, age, and student year groups. We also assessed motivation improvement and perception of knowledge pre and post-conference. We finally compared motivation towards oncology between students who attended the conference (pre and post-conference) versus the national average.

### Outcomes

#### National Survey

We assessed students’ views towards oncology as measured by Likert scale responses concerning their interest, knowledge and exposure, and teaching satisfaction.

#### Conference feedback questionnaire

The effectiveness of the conference as an educational, networking and motivational tool was measured by assessing for a significant change (*p* < 0.05) in students’ pre and post-conference responses.

### Ethical approval

All surveys were prospectively approved by the Queen Mary University of London Ethics Committee (Reference number QMREC2348). All participants consenting to anonymous data being used for this study and/ or additional data analysis and publication via a peer-reviewed journal.

### Procedures to minimise bias

Questionnaires were piloted in a small group of medical students before being reviewed and launched to ensure questions were unambiguous and phrasing did not generate responder bias. Informed consent statements were included at the beginning of each survey. The national survey gathered completely anonymous data, hence, participants were informed of their right to withdraw only prior to survey submission as data could not be selected for exclusion thereafter. Conversely, in the pre and post-conference surveys, participants provided an anonymised identifier (ID) that allowed for matching of individual participant data to enable comparison of results and view changes in perception. Conference participants were informed of their right to withdraw at any time even after survey submission as responses could be selected for exclusion by quoting the anonymised ID.

### Data collection

All surveys were conducted via a standardised, web-based, data collection form (Google Form, Google, Alphabet Inc.). Responses were saved to a password-protected Excel file. Data was cleaned and matched by S.H. and K.R. for ease of statistical analysis. Regarding free text responses (qualitative data) S.H. and K.R. proceeded to a pilot thematic analysis; this resulted in discrete thematic axes. Those thematic axes were revised by N.S. to ensure accuracy of data; any discrepancy was resolved through discussion with the senior author of the study.

### Statistical analysis

Statistical analysis was completed on IBM SPSS Statistics software for Windows Version 26 and GraphPad Prism 8 software. SPSS descriptive statistics were used to analyse student demographics, views on undergraduate oncology teaching, and conference feedback. Following assessment of data distribution, we used Mann-Whitney U (MWU) test for different group comparisons and Wilcoxon signed-rank (WSR) test for paired association. *P*-value less than 0.05 was considered as statistically significant. Cronbach’s Alpha coefficient and the Corrected Item-total Correlations (ICC) assessed the internal consistency reliability of national survey data. Cronbach’s Alpha between 0.5 and 0.7 was considered to reflect an acceptable internal consistency while > 0.7 was good. The ICC determined the level of agreement between measurements. ICC < 0.2 was regarded as poor agreement, 0.21–0.40 as fair, 0.61–0.80 as good, and 0.81–1.0 as very good [25].

## Results

### Demographics

#### National survey

In total, 166 medical students completed the national survey. The median age of responders was 22 (IQR = 3). The majority (82%) studied on an undergraduate course while the remainder either studied on a graduate entry programme (GEP), which is an accelerated medical programme for students with a previous degree, or were taking a year out of their medical course to intercalate in another field of study obtaining an additional degree. Responders included students of all year groups, representing 22 different UK medical schools. Participant demographics are summarised in Table 1.

**Table 1:** University affiliation and year of study of the national survey (*n* = 166) and conference cohorts (*n* = 34)

	National survey^a^	Conference^a^
University		
Barts and the London School of Medicine and Dentistry	35 (21.1)	15 (44.1)
Birmingham University	1 (0.6)	1 (2.9)
Cambridge University	1 (0.6)	2 (5.9)
Cardiff University	4 (2.4)	–
Dundee University	7 (4.2)	–
Edinburgh University	3 (1.8)	–
Exeter University	14 (8.4)	–
Glasgow University	14 (8.4)	–
Imperial College London	2 (1.2)	2 (5.9)
King’s College London	4 (2.4)	8 (23.5)
Leeds University	3 (1.8)	–
Liverpool University	16 (9.6)	1 (2.9)
Manchester University	3 (1.8)	–
Newcastle University	2 (1.2)	–
Norwich Medical School (University of East Anglia)	13 (7.8)	–
Plymouth University	2 (1.2)	2 (5.9)
Southampton University	19 (11.4)	–
St Andrews University	3 (1.8)	–
St George’s, University of London	5 (3.0)	1 (2.9)
Swansea University	10 (6.0)	–
University College London	3 (1.8)	1 (2.9)
Warwick University	2 (1.2)	1 (2.9)
Year of study		
GEP^d^ year 1	4 (2.4)	–
GEP year 2	1 (0.6)	–
GEP year 3	2 (1.2)	–
GEP year 4	3 (1.8)	–
Intercalating between year 2 and 3	3 (1.8)	–
Intercalating between year 3 and 4	13 (7.8)	5 (14.7)
Intercalating between year 4 and 5	4 (2.4)	1 (2.9)
MBBS^e^ year 1	14 (8.4)	5 (14.7)
MBBS year 2	37 (22.3)	9 (26.5)
MBBS year 3	33 (19.9)	8 (23.5)
MBBS year 4	31 (18.7)	5 (14.7)
MBBS year 5	21 (12.7)	1 (2.9)
Previous degree		
No previous degree	103 (62.0)	21 (61.8)
Previous degree	63 (38.0)	13 (38.2)
Undergraduate degree^b^	56 (33.7)	12 (35.3)
Postgraduate degree^c^	16 (9.6)	5 (14.7)
2 previous degrees	9 (5.4)	4 (11.8)

^a^ Data given as number of students (%)

^b^ Undergraduate degrees included BA, BMedSc, BSc, BEng

^c^ Postgraduate degrees included MRes, MSc, MA, MPharm

^d^ Graduate Entry Programme (GEP)

^e^ Bachelor of Medicine, Bachelor of Surgery (MBBS)

#### Conference

Sixty-two students attended the conference, out of which 58 (93.5%) completed the pre-conference questionnaire and 41 (66.1%) completed the post-conference questionnaire. Comparative statistics were performed on 34 (54.8%) medical students for whom pre and post-conference data was available. Of these 34, the median age was 21 (IQR = 1.5) (Table 1).

### Students’ views on oncology

Table 2 describes students’ views towards oncology compared to those attending the conference (prior and after). In a nutshell, nationally, most students report being unlikely or unsure towards pursuing a career in oncology (*mean score*: 2.7 ± 1.1) prior to entering medical school, and neutral interest at the time of the conference (*mean score*: 3.1 ± 1.2) (Table 2). Overall, students perceive oncology as a challenging specialty (*mean score*: 4.5 ± 0.7) and report minimal exposure during their undergraduate training (*mean score*: 1.9 ± 1.0) (Table 2).

**Table 2:** Views on oncology: responses from the national survey (*n* = 166) as well as both pre- and post-conference questionnaires (*n* = 34)

Question	National survey^a^	Pre-conference^a^	p-value^c^	Post-conference^a^	p-value^d^
How would you rate your interest in oncology^b^					
prior to entering medical school?	2.7 ± 1.1			3.4 ± 1.6	0.0044
at this point in time?	3.1 ± 1.2			4.0 ± 0.9	< 0.0001
How likely are you to pursue a career in oncology?	2.7 ± 1.1	3.1 ± 1.2	0.0322	3.9 ± 0.8	< 0.0001
Rate your interest in the following oncology career pathways					
Clinical research (e.g. clinical trials)	2.9 ± 1.3	3.6 ± 1.2	0.0044	3.8 ± 1.0	< 0.0001
Scientific research (e.g. laboratory, pre-clinical)	2.3 ± 1.3	3.2 ± 1.0	< 0.0001	3.5 ± 1.1	< 0.0001
Clinical oncology	3.4 ± 1.1	3.5 ± 1.1		3.8 ± 0.9	
Medical oncology	3.5 ± 1.1	3.6 ± 1.0		3.7 ± 1.1	
Surgical oncology	2.8 ± 1.3	3.2 ± 1.3		3.4 ± 1.3	0.0173*
Palliative care	2.9 ± 1.3	2.6 ± 1.3		2.8 ± 1.3	
Rate how much you agree with the following statements					
Oncology is a challenging specialty.	4.5 ± 0.7	4.1 ± 1.0	0.0171	4.1 ± 0.7	0.0051
If I were an oncologist, I am afraid that I would be overly sensitive.	3.3 ± 1.2	3.1 ± 1.0		2.9 ± 1.0	0.0494*
If I were an oncologist, I am afraid that I would be too thick-skinned.	2.2 ± 1.0	2.1 ± 1.0		2.0 ± 0.7	
If I were an oncologist, I feel like I would be able to cope with the emotional challenges in this field.	3.2 ± 1.0	3.3 ± 0.8		3.8 ± 0.7	0.0058
I am overall optimistic about cancer as a whole.	3.1 ± 1.0	3.4 ± 1.2		3.7 ± 1.0	0.0015*
I am overall pessimistic about cancer as a whole.	2.7 ± 1.0	2.3 ± 1.1	0.0232	2.1 ± 1.0	0.0035
Rate how much knowledge or exposure you have had in the following aspects of oncology					
Career and specialty training pathway	1.9 ± 1.0	2.5 ± 1.2	0.0070	3.4 ± 1.0	< 0.0001
Patient pathway (from diagnosis to treatment to recovery)	2.8 ± 1.2	3.4 ± 1.1	0.0201	3.9 ± 1.0	< 0.0001
Patient experience and views	3.0 ± 1.1	3.1 ± 1.0		4.0 ± 1.0	< 0.0001
Types of cancer research and how they are carried out	2.1 ± 1.1	2.7 ± 1.1	0.0052	3.6 ± 1.0	< 0.0001
Understanding of the different multi-disciplinary members and their roles in the cancer pathway	3.1 ± 1.3	3.6 ± 1.1		4.0 ± 0.8	0.0001
Rate your confidence in the following					
Communicating with a cancer patient	3.0 ± 1.1	3.0 ± 1.0		3.8 ± 0.8	< 0.0001
Speaking about death and dying with a cancer patient	2.3 ± 1.1	2.1 ± 1.1		3.0 ± 1.0	0.0005
Identifying skin cancer lesions	2.4 ± 1.2	1.7 ± 0.9	0.0017	3.3 ± 0.8	< 0.0001
Knowledge of the organisation and important aspects that govern clinical trial research	2.1 ± 1.1	2.3 ± 1.1		3.6 ± 1.1	< 0.0001
How to build your CV towards a career in oncology	1.8 ± 1.0	2.3 ± 0.8	< 0.0001	3.6 ± 1.1	< 0.0001
Knowledge of the role of interventional radiology in diagnosing and treating cancer	2.4 ± 1.1	2.4 ± 1.1		3.8 ± 1.0	< 0.0001

^a^ Data is reported as the mean value of the Likert score ± standard deviation

^b^ Question was only asked in the post-conference questionnaire

^c^ *p*-value obtained from MWU analysis between national survey and pre-conference questionnaire

^d^ *p*-value obtained from MWU analysis between national survey and post-conference questionnaire

* p-value < 0.05 achieved when comparing national mean to post-conference mean and not significant when comparing pre-conference mean to post-conference mean (Table 4) due to small sample size

### Comparison to conference delegates’ views

Conference attendees reported a statistically significant higher likelihood of pursuing a career in oncology compared to national responders (3.1 vs. 2.7/5, *p* = 0.0322, Table 2), however, overall most questionnaire items had similar responses. Noteworthily, delegates did not consider oncology to be as challenging (4.1 vs. 4.5/5, *p* = 0.0171), as well as expressed less pessimism towards cancer (2.3 vs. 2.7/5, *p* = 0.0232). Additionally, delegates reported higher exposure to oncology training pathways (2.5 vs. 1.9/5, *p* = 0.0070), the cancer patient pathway (3.4 vs. 2.8/5, *p* = 0.0201), cancer research (2.7 vs. 2.1/5, *p* = 0.0052), and more confidence in building their CVs towards a career in oncology (2.3 vs. 1.8/5, *p* < 0.0001).

### Satisfaction with undergraduate oncology teaching

Nationally, students reported poor satisfaction with pre-clinical and clinical oncology teaching (*mean score*: 2.8 ± 1.1, Table 3). Most students estimated they would receive between 1 and 2 weeks (21.7%) or 3–4 weeks (15.7%) of dedicated oncology teaching throughout their degree (Supplementary Table 1); median 3–4 weeks.

**Table 3:** Teaching satisfaction: comparison of responses from the national survey (*n* = 166) and post-conference questionnaire (*n* = 34)

Question	National survey^a^	Post-conference^a^	p-value^b^
How satisfied are you with the quality of pre-clinical oncology teaching at your university			
Teaching hours	2.8 ± 1.1	3.3 ± 1.0	0.0120
Content and material	2.9 ± 1.1	3.4 ± 1.0	0.0084
Structure of the course	2.8 ± 1.0	3.2 ± 1.2	0.0410
How satisfied are you with the quality of clinical oncology teaching at your university			
Teaching hours	2.8 ± 1.1	2.9 ± 1.0	
Content and material	2.8 ± 1.1	2.9 ± 0.9	
Structure of the course	2.8 ± 1.1	2.9 ± 1.0	

^a^ Data is reported as the mean value of the Likert score ± standard deviation

^b^ p-value obtained from MWU test analysis between national survey and post-conference questionnaire

Delegates reported similar teaching satisfaction ratings to national survey responders, yet pre-clinical teaching satisfaction was statistically significantly higher (Table 3). Conference attendees also reported more dedicated oncology teaching hours, reporting 4–6 weeks (14.7%) or 6–8 weeks (14.7%) of teaching (Supplementary Table 1); median 4–6 weeks.

### Impact of oncology conference

Table 4 represents the impact of the oncology conference on students’ views.

**Table 4:** Views on oncology: comparison of responses from pre- and post-conference questionnaires (*n* = 34)

Question	Pre-conference	Post-conference	p-value
How likely are you to pursue a career in oncology?	3.1 ± 1.2	3.9 ± 0.8	0.0012
Rate your interest in the following oncology career pathways			
Clinical research (e.g. clinical trials)	3.6 ± 1.2	3.8 ± 1.0	
Scientific research (e.g. laboratory, pre-clinical)	3.2 ± 1.0	3.5 ± 1.1	
Clinical oncology	3.5 ± 1.1	3.8 ± 0.9	
Medical oncology	3.6 ± 1.0	3.7 ± 1.1	
Surgical oncology	3.2 ± 1.3	3.4 ± 1.3	
Palliative care	2.6 ± 1.3	2.8 ± 1.3	
Rate how much you agree with the following statements			
Oncology is a challenging specialty.	4.1 ± 1.0	4.1 ± 0.7	
If I were an oncologist, I am afraid that I would be overly sensitive.	3.1 ± 1.0	2.9 ± 1.0	
If I were an oncologist, I am afraid that I would be too thick-skinned.	2.1 ± 1.0	2.0 ± 0.7	
If I were an oncologist, I feel like I would be able to cope with the emotional challenges in this field.	3.3 ± 0.8	3.8 ± 0.7	0.0278
I am overall optimistic about cancer as a whole.	3.4 ± 1.2	3.7 ± 1.0	
I am overall pessimistic about cancer as a whole.	2.3 ± 1.1	2.1 ± 1.0	
Rate how much knowledge or exposure you have had in the following aspects of oncology			
Career and specialty training pathway	2.5 ± 1.2	3.4 ± 1.0	< 0.0001
Patient pathway (from diagnosis to treatment to recovery)	3.4 ± 1.1	3.9 ± 1.0	0.0008
Patient experience and views	3.1 ± 1.0	4.0 ± 1.0	< 0.0001
Types of cancer research and how they are carried out	2.7 ± 1.1	3.6 ± 1.0	< 0.0001
Understanding of the different multi-disciplinary members and their roles in the cancer pathway	3.6 ± 1.1	4.0 ± 0.8	0.0348
Rate your confidence in the following			
Communicating with a cancer patient	3.0 ± 1.0	3.8 ± 0.8	< 0.0001
Speaking about death and dying with a cancer patient	2.1 ± 1.1	3.0 ± 1.0	< 0.0001
Identifying skin cancer lesions	1.7 ± 0.9	3.3 ± 0.8	< 0.0001
Knowledge of the organisation and important aspects that govern clinical trial research	2.3 ± 1.1	3.6 ± 1.1	< 0.0001
How to build your CV towards a career in oncology	2.3 ± 0.8	3.6 ± 1.1	< 0.0001
Knowledge of the role of interventional radiology in diagnosing and treating cancer	2.4 ± 1.1	3.8 ± 1.0	< 0.0001

^a^ Data is reported as the mean value of the Likert score ± standard deviation

^b^ *p*-value obtained from WSR test analysis between pre- and post-conference questionnaires

#### Generating interest in oncology and shaping students’ views

Following the symposium, students reported a statistically significant increase in overall likelihood of pursuing a career in oncology (3.9 vs. 3.1/5, *p* = 0.0012) and improved ability to cope with the emotional challenges associated with a career in oncology (3.8 vs. 3.3/5, *p* = 0.0278) which the conference aimed to address through the patient panel and patient interaction workshop.

#### Value of conference as an educational tool

Post-conference, students felt a significant improvement in their knowledge and confidence concerning all aspects discussed during the lectures and workshops (Table 4).

### Internal consistency reliability analysis

The Cronbach’s Alpha coefficient based on standardised items was 0.849 (Cronbach’s Alpha coefficient = 0.849) demonstrating good internal consistency of the survey data. The mean ICC was 0.226 indicating fair agreement between measurements.

## Discussion

### National findings

The national survey aimed to determine medical students’ views on oncology including their knowledge, confidence in skills, and teaching satisfaction to identify areas of improvement in undergraduate education. Lack of interest and insufficient exposure to oncology including the specialty training pathway was identified. Oncology was rated as a challenging specialty, and students reported poor confidence in oncology skills especially in regard to building their CV towards a career in oncology; understanding clinical trial research; communicating about death with patients; identifying skin cancers; and understanding the role of interventional radiology in cancer treatment (Table 2). Students were unsatisfied with oncology teaching. Findings are consistent worldwide, with students reporting limited oncology specialty exposure [9, 27]; teaching dissatisfaction [28]; and poor confidence in oncology care [9, 29, 30]. Newly-qualified doctors report limited undergraduate exposure to cancer patients, and lack of cancer care knowledge [6, 31]. Furthermore, although recommendations for undergraduate oncology teaching indicate at least 2 weeks of dedicated teaching, [32, 33] with 2–3-weeks full-time towards the end of undergraduate years, [14] most national survey responders (20.7%) estimated receiving only 1–2 weeks of teaching. A third of students (33.1%) were unable to estimate the number of teaching weeks raising questions about whether these students receive any dedicated oncology teaching at all. This corroborates with other research, suggesting only 61% of first-year trainee doctors receive any oncology placement at medical school, [6] while 36% of UK medical schools offer dedicated oncology placements [10].

### Conference findings

We hypothesised that the conference may be a valuable educational tool. Delegates’ inclination towards oncology career pathways was further increased post-conference. The event successfully increased students’ knowledge and exposure to oncology including the specialty training and patient pathways. The educational utility of the conference is demonstrated by delegates’ dramatically improved confidence in skills, especially in identifying skin cancer lesions. Indeed, extracurricular teaching programmes including mentorship schemes, [32–35] conferences, and dedicated teaching courses [15, 18–21] are effective in generating student interest while improving knowledge and skills.

### Significance of findings

With increasing global demand for oncologists to join the workforce, [7, 8] the importance of early undergraduate oncology teaching cannot be overemphasised. The majority of cancer patient care is performed in specialist centres, hence limited student oncology exposure may be due to restricted placement availability [16]. Nonetheless, placement accessibility is of utmost importance as early specialty exposure increases medical students’ interest [34–36]. Thus, lack of student interest in oncology is unsurprising considering their limited exposure.

### Recommendations

A targeted educational needs assessment of medical students is essential in devising guidelines for an optimal undergraduate oncology curriculum [37]. Our recommendations are based on students’ feedback in free text responses. Nationally, students advocated for more dedicated clinical exposure to oncology, increased teaching hours, and more diverse coverage of cancer topics. Several students suggested restructuring oncology curricula and more clinical skills teaching covering breaking bad news and communicating with terminally-ill patients. Developing guidelines encompassing core oncology skills-based knowledge outcomes is a priority. Familiarising students with a specialty is an effective strategy to prevent specialty attrition and trainee burn-out. Increasing students’ oncology exposure is a key way to generate interest and motivate students to pursue this specialty in hopes of creating a better future generation of holistically qualified oncologists who are enthusiastic about laboratory and clinical research [38–40]. Although securing clinical placements for students at specialist oncologist centres may be difficult to achieve, a lot of cancer patient management is carried out in non-specialist centres including primary care. Hence, encompassing more oncology teaching within general practice placements would be a helpful means to overcome this issue. Being able to break bad news sensitively is relevant for all aspects of medicine and more dedicated communication skills teaching on the subject is essential.

A criticism of undergraduate oncology teaching is that it can at times be pitched to a postgraduate level, thus, contributing to disinterest and confusion amongst students. Medical school teaching should be tailored to preparing students to manage common oncological presentations and emergencies that they will encounter as foundation year doctors, as opposed to specific cancer treatment regimens. The clinical oncology faculty of the Royal College of Radiologists (RCR) and the Royal College of Physicians (RCP) jointly published the “Undergraduate non-surgical oncology curriculum” in September 2020 [41] in accordance with the latest General Medical Council “Outcomes for graduates 2018”. UK medical schools often have their own curriculum with intended learning outcomes for oncology and these should be continually scrutinised and revised to meet the requirements set out by the RCR and RCP curriculum. Some of the salient outcomes that deserve more emphasis include:

- “Undertake a focused oncological history focusing on common and ‘red flag’ symptoms”
- “Undertake a focused oncological examination e.g. assessment for metastatic spinal cord compression”
- “Describe appropriate urgent referral pathways for patients with a suspected cancer”
- “Demonstrate communication skills e.g. principles of breaking bad news and shared decision-making with a patient”
- “Demonstrate a holistic clinical assessment of patients with cancer including the local and systemic sequelae of common cancer presentations”.

Many of these are also echoed by the students’ responses in our surveys.

### Strengths

Scarce data is available on medical students’ perspectives on current undergraduate oncology teaching with data almost non-existent for the UK [28, 42–47]. To our knowledge, this is the first national cross-sectional survey of UK medical students’ views in oncology. The strengths of this study include its prospective protocol approval, the national representability of findings to several medical schools, and the variety of questionnaire components capturing different angles.

### Limitations

#### National findings

Limitations of this study include the small sample size and lack of questionnaire validation. A sample size of >1000 national responders would have increased robustness of results. Representativeness of findings was limited by only having responses from 22 UK medical schools. Lack of response from certain institutions may be explained by small cohort sizes in these medical schools and ‘survey fatigue’. A large proportion of student responders were in pre-clinical years and will inevitably have been less confident in communicating with cancer patients. These students may not be aware of clinical oncology teaching if this was scheduled to take place in clinical years, hence providing an inaccurate underestimation of the amount of dedicated oncology teaching they receive. Although we captured some data regarding students’ satisfaction with pre-clinical oncology teaching (Table 3), our questionnaires primarily focused on clinical teaching. Pre-clinical oncology teaching is vastly inconsistent between medical schools and is challenging to assess as it is often integrated within pathology, histology and organ systems’ teaching.

#### Conference findings

With regards to the effectiveness of the conference as an educational tool, the overall increase in students’ self-reported knowledge, exposure, and confidence was not validated by an objective method of assessment. The apparent changes could have been influenced by response acquiescence or acceptance bias which led students to provide a higher estimate of their knowledge in the post-conference questionnaire. Resurveying students after a longer period of time (e.g. 12 months) may eliminate these short-lived artifacts. We could also examine if delegates carried on this confidence into their foundation training posts and compare their average scores with those of foundation doctors who had not attended the event. Lastly, we cannot discount the inherent selection bias as students who chose to attend the conference already had interest towards oncology and an innate inclination towards this specialty. This must be considered when interpreting our findings, especially when conference results are compared or generalised to the national cohort.

## Conclusion

In summary, findings herein demonstrate deficiencies in medical students’ knowledge, exposure, and confidence in skills especially in regard to building their CV towards a career in oncology; understanding clinical trial research; communicating about death with patients; identifying skin cancers; and understanding the role of interventional radiology in cancer treatment. Students estimate receiving less than the recommended 2–3 weeks of oncology teaching [14]. Although the undergraduate oncology conference has demonstrated effectiveness as an educational tool, it is still necessary to revisit and strive to improve undergraduate oncology curricula. Developing a high quality uniformly structured, systematic undergraduate oncology curriculum should become a national and international priority; equally, proposed teaching guidelines should be revised and adhered to. Students’ attitudes should be considered when formulating curricula as they provide valuable insight into future doctors’ needs.

## Supplementary Information

**Additional file 1.** Appendix 1. National survey.

**Additional file 2.** Appendix 2. Pre-conference questionnaire.

**Additional file 3.** Appendix 3. Post-conference questionnaire.

**Additional file 4.** Appendix 4. Ethical approval.

**Additional file 5. **Supplementary Tables.

## Data Availability

The datasets/ questionnaires used and analysed during the current study are not publicly available due to data protection reasons but are available from the corresponding author on reasonable request.
