# Supplementary material for: Inspiring the future generation of oncologists: a UK-wide study of medical students’ views towards oncology
Source: BMC Med Educ. 2021 Feb 2;21:82. doi: 10.1186/s12909-021-02506-0 (PMC7852146; doi:10.1186/s12909-021-02506-0)
Supplement: Supplementary file 1 — Additional file 1. Appendix 1. National survey. [file 12909_2021_2506_MOESM1_ESM.pdf]

National Medical Student Oncology Survey 2020

* Required

Project description and consent statement

Dear Medical Student,

We would like to invite you to fill in this questionnaire as part of a research project aimed at understanding students' views on oncology. Please read the following to gain a better understanding of what this will involve, should you choose to participate.

We would like to understand who you are as a participant, and what your existing perceptions of oncology are. This will include some basic questions about your educational background followed by a series of questions about your thoughts and feelings on oncology and its different sub-specialties, and how your medical school teaches this subject, should this be applicable. We will not collect your name or any other personal information that will make you identifiable. This questionnaire will take approximately 5-10 minutes.

By completing the survey, you will help us understand what students currently enjoy about oncology teaching and what they think is lacking and how the curriculum can improve.

A few important points:
- We intend on publishing these results in an academic journal, however your answers will be kept confidential and your responses will be completely anonymous.
- The data we collect will not be used for any other purpose other than those stated above.
- You have the right to withdraw before you submit the survey if you wish to do so for any reason.
- Please note that after you submit your responses it is not possible to withdraw your information as your responses are anonymous and cannot be selected for exclusion.

If you have any questions please contact ha16849@qmul.ac.uk.

1. I have read and understood the above information and I voluntarily consent to participate in this study. *

Mark only one oval.

- ( ) Yes
- ( ) No

Part 1: Participant demographics

Please be honest, all answers are anonymised!

2. Age *

____

3. Please specify your year of study *

If your medical curriculum is a 6-year curriculum, and you are in a year that is your project/ BSc/ non-medicine year, please consider this an "intercalating" year.

Mark only one oval.

- MBBS year 1
- MBBS year 2
- MBBS year 3
- MBBS year 4
- MBBS year 5
- Intercalating between year 1 and 2
- Intercalating between year 2 and 3
- Intercalating between year 3 and 4
- Intercalating between year 4 and 5
- Other: ______________________

4. If you are a student please state your University of study *

____

5. Please indicate if you have completed any other degrees. *

Check all that apply.

- [ ] I have not completed any other degrees
- [ ] BA
- [ ] BSc
- [ ] MSc/MA
- [ ] PhD
- Other: [ ] ______________________

Part 2: Views on oncology

This section is for us to understand how you personally perceive oncology as a specialty. Please be honest, all answers are anonymised!

6. How likely are you to pursue a career in oncology? *

1= Very unlikely, 2= Unlikely, 3= Unsure, 4= Likely, 5=Very likely

Mark only one oval.

	1	2	3	4	5	
Very unlikely	⬭	⬭	⬭	⬭	⬭	Very likely

7. Rate your interest in the following oncology career pathways. *

1= Very uninterested, 2= Uninterested, 3= Neutral, 4= Interested, 5= Very interested

Mark only one oval per row.

	1	2	3	4	5
Clinical research (e.g. clinical trials)	⬭	⬭	⬭	⬭	⬭
Scientific research (e.g. laboratory, pre-clinical)	⬭	⬭	⬭	⬭	⬭
Clinical oncology	⬭	⬭	⬭	⬭	⬭
Medical oncology	⬭	⬭	⬭	⬭	⬭
Surgical oncology	⬭	⬭	⬭	⬭	⬭
Palliative care	⬭	⬭	⬭	⬭	⬭

8. Rate how much you agree with the following statements. *

1= Highly disagree, 2= Somewhat disagree, 3= Neutral, 4= Somewhat agree, 5= Highly agree

Mark only one oval per row.

	1	2	3	4	5
Oncology is a challenging specialty.	◯	◯	◯	◯	◯
If I were an oncologist, I am afraid that I would be overly sensitive.	◯	◯	◯	◯	◯
If I were an oncologist, I am afraid that I would be too thick-skinned.	◯	◯	◯	◯	◯
If I were an oncologist, I feel like I would be able to cope with the emotional challenges in this field.	◯	◯	◯	◯	◯
I am overall optimistic about cancer as a whole.	◯	◯	◯	◯	◯
I am overall pessimistic about cancer as a whole.	◯	◯	◯	◯	◯

9. Rate how much knowledge or exposure you have had in the following aspects of oncology. *

1= Very little/ none, 2= Minimal, 3= Some, 4= A fair amount, 5= A lot

Mark only one oval per row.

	1	2	3	4	5
Career and specialty training pathway	◯	◯	◯	◯	◯
Patient pathway (from diagnosis to treatment to recovery)	◯	◯	◯	◯	◯
Patient experience and views	◯	◯	◯	◯	◯
Types of cancer research and how they are carried out	◯	◯	◯	◯	◯
Understanding of the the different multi-disciplinary members and roles in the cancer pathway	◯	◯	◯	◯	◯

10. Rate your confidence in the following: *

1= Not confident at all, 2= Minimally confident, 3= Somewhat confident, 4= Quite confident, 5= Very confident

Mark only one oval per row.

	1	2	3	4	5
Communicating with a cancer patient	◯	◯	◯	◯	◯
Speaking about death and dying with a cancer patient	◯	◯	◯	◯	◯
Identifying skin cancer lesions	◯	◯	◯	◯	◯
Knowledge of the organisation and important aspects that govern clinical trial research	◯	◯	◯	◯	◯
How to build your CV towards a career in oncology	◯	◯	◯	◯	◯
Knowledge of the role of interventional radiology in diagnosing and treating cancer	◯	◯	◯	◯	◯

Part 3: Views on oncology in your medical school curriculum

Please be honest, all answers are anonymised!

11. How would you rate your interest in oncology prior to entering medical school? *

1= Very uninterested, 2= Uninterested, 3= Neutral, 4= Interested, 5= Very interested

Mark only one oval.

	1	2	3	4	5	
Very uninterested	◯	◯	◯	◯	◯	Very interested

12. How would you rate your interest in oncology at this point in time? *

1= Very uninterested, 2= Uninterested, 3= Neutral, 4= Interested, 5= Very interested

Mark only one oval.

	1	2	3	4	5	
Very uninterested						Very interested

13. How satisfied are you with the quality of pre-clinical oncology teaching at your university? *

1= Very dissatisfied, 2= Somewhat dissatisfied, 3= Neutral, 4= Satisfied, 5= Very satisfied

Mark only one oval per row.

	1	2	3	4	5	N/A
Teaching hours						
Content and material						
Structure of the course						

14. How satisfied are you with the quality of clinical oncology teaching at your university? *

1= Very dissatisfied, 2= Somewhat dissatisfied, 3= Neutral, 4= Satisfied, 5= Very satisfied

Mark only one oval per row.

	1	2	3	4	5	N/A
Teaching hours						
Content and material						
Structure of teaching						

15. How many weeks of mandatory oncology teaching does your medical curriculum provide? Please provide an estimate of the total number weeks over the FULL duration of your medical degree. *

Mark only one oval.

- <1 week
- 1-2 weeks
- 3-4 weeks
- 4-6 weeks
- 6-8 weeks
- 8-10 weeks
- 10-12 weeks
- >12 weeks
- Not sure
- Other: ____________________

16. How do you think oncology teaching can be improved within your curriculum? *

____
____
____
____
____

End of questionnaire

Thank you for completing the National Medical Student Oncology Survey 2020.

All answers are anonymised and will be used only for the purposes described in the consent statement.

Please note that after you submit your responses it is not possible to withdraw your information as your responses are anonymous and cannot be selected for exclusion.

If you have any questions, please email ha16849@qmul.ac.uk

This content is neither created nor endorsed by Google.

Google Forms
