# Supplementary material for: Inspiring the future generation of oncologists: a UK-wide study of medical students’ views towards oncology
Source: BMC Med Educ. 2021 Feb 2;21:82. doi: 10.1186/s12909-021-02506-0 (PMC7852146; doi:10.1186/s12909-021-02506-0)
Supplement: Supplementary file 2 — Additional file 2. Appendix 2. Pre-conference questionnaire. [file 12909_2021_2506_MOESM2_ESM.pdf]

NUOC 2020 Pre-Conference Questionnaire

* Required

Project description and consent statement

Dear Participant,

As a part of the National Undergraduate Oncology Conference 2020, we would like to invite you to fill in this pre-conference questionnaire. Please read the following to gain a better understanding of what this will involve, should you choose to participate.

As this is the first time an oncology conference of this scale is held, we would like to understand who you are as a participant, and what your existing perceptions of oncology are. This will include some basic questions about your educational background followed by a series of questions about your thoughts and feelings on oncology and its different sub-specialties, and how your medical school teaches this subject, should this be applicable. We will not collect your name or any other personal information that will make you identifiable. This questionnaire will take approximately 5-10 minutes. You will require your conference registration number or generated random ID to complete this questionnaire.

After the conference, we will also send you a post-conference questionnaire. Note that by completing the pre-conference questionnaire you are not obliged to complete the post-conference questionnaire, although it is encouraged that you do so.

By completing the survey, you will help us understand what people are interested in, how to improve future conferences, and if the conference has made any impact or generated any new interest.

A few important points:
- We intend on publishing these results in an academic journal, however your answers will be kept confidential and your responses will be completely de-identified (i.e. all possible identifying characteristics, such as your conference ID, will be separated from your answers and any publicly available data).
- The data we collect will not be used for any other purpose other than those stated above.
- You have the right to withdraw at any time, before or after you submit the questionnaire, if you wish to do so for any reason. You will need to quote your conference registration number or generated random ID to withdraw your response after submission.

If you have any questions please contact ha16849@qmul.ac.uk.

1. I have read and understood the above information and I voluntarily consent to participate in this study. *

Mark only one oval.

- ⬭ Yes
- ⬭ No

2. Please provide your conference registration number *

This will be used to match your responses to the pre- and post-conference questionnaires. You may withdraw your data after completing the questionnaire by quoting this number. If you do not have a conference registration number, please generate a random ID by putting the last 4 digits of your student ID number and first 2 letters on your surname (e.g. if your student number is 123456789 and your surname is Smith, your generated random ID is 6789SM)

____

Part 1: Participant demographics

Please be honest, all answers are de-identified!

3. Age *

____

4. Occupation *

Mark only one oval.

- ( ) Medical student
- ( ) University student of other degree
- ( ) Medical professional
- ( ) Other: ______________________________

5. Please specify your year of study *

If your medical curriculum is a 6-year curriculum, and you are in a year that is your project/ BSc/ non-medicine year, please consider this an "intercalating" year.

Mark only one oval.

- MBBS year 1
- MBBS year 2
- MBBS year 3
- MBBS year 4
- MBBS year 5
- Intercalating between year 1 and 2
- Intercalating between year 2 and 3
- Intercalating between year 3 and 4
- Intercalating between year 4 and 5
- Other: ____________

6. If you are a student please state your University of study *

____

7. Please indicate if you have completed any other degrees. *

Check all that apply.

- [ ] I have not completed any other degrees
- [ ] BA
- [ ] BSc
- [ ] MSc/MA
- [ ] PhD

Other: [ ] ____________

Part 2: Views on oncology

This section is for us to understand how you personally perceive oncology as a specialty. Please be honest, all answers are de-identified!

8. How likely are you to pursue a career in oncology? *

1= Very unlikely, 2= Unlikely, 3= Unsure, 4= Likely, 5=Very likely

Mark only one oval.

	1	2	3	4	5	
Very likely	⬭	⬭	⬭	⬭	⬭	Very unlikely

9. Rate your interest in the following oncology career pathways. *

1= Very uninterested, 2= Uninterested, 3= Neutral, 4= Interested, 5= Very interested

Mark only one oval per row.

	1	2	3	4	5
Clinical research (e.g. clinical trials)	⬭	⬭	⬭	⬭	⬭
Scientific research (e.g. laboratory, pre-clinical)	⬭	⬭	⬭	⬭	⬭
Clinical oncology	⬭	⬭	⬭	⬭	⬭
Medical oncology	⬭	⬭	⬭	⬭	⬭
Surgical oncology	⬭	⬭	⬭	⬭	⬭
Palliative care	⬭	⬭	⬭	⬭	⬭

10. Rate how much you agree with the following statements. *

1= Highly disagree, 2= Somewhat disagree, 3= Neutral, 4= Somewhat agree, 5= Highly agree

Mark only one oval per row.

	1	2	3	4	5
Oncology is a challenging specialty.	◯	◯	◯	◯	◯
If I were an oncologist, I am afraid that I would be overly sensitive.	◯	◯	◯	◯	◯
If I were an oncologist, I am afraid that I would be too thick-skinned.	◯	◯	◯	◯	◯
If I were an oncologist, I feel like I would be able to cope with the emotional challenges in this field.	◯	◯	◯	◯	◯
I am overall optimistic about cancer as a whole.	◯	◯	◯	◯	◯
I am overall pessimistic about cancer as a whole.	◯	◯	◯	◯	◯

11. Rate how much knowledge or exposure you have had in the following aspects of oncology. *

1= Very little/ none, 2= Minimal, 3= Some, 4= A fair amount, 5= A lot

Mark only one oval per row.

	1	2	3	4	5
Career and specialty training pathway	◯	◯	◯	◯	◯
Patient pathway (from diagnosis to treatment to recovery)	◯	◯	◯	◯	◯
Patient experience and views	◯	◯	◯	◯	◯
Types of cancer research and how they are carried out	◯	◯	◯	◯	◯
Understanding of the the different multi-disciplinary members and their roles in the cancer pathway	◯	◯	◯	◯	◯

12. Rate your confidence in the following: *

1= Not confident at all, 2= Minimally confident, 3= Somewhat confident, 4= Quite confident, 5= Very confident

Mark only one oval per row.

	1	2	3	4	5
Communicating with a cancer patient					
Speaking about death and dying with a cancer patient					
Identifying skin cancer lesions					
Knowledge of the organisation and important aspects that govern clinical trial research					
How to build your CV towards a career in oncology					
Knowledge of the role of interventional radiology in diagnosing and treating cancer					

Part 3: Views on oncology in your medical school curriculum

Please be honest, all answers are de-identified!

13. How would you rate your interest in oncology prior to entering medical school? *

1= Very uninterested, 2= Uninterested, 3= Neutral, 4= Interested, 5= Very interested

Mark only one oval.

	1	2	3	4	5	
Very uninterested						Very interested

14. How would you rate your interest in oncology at this point in time? *

1= Very uninterested, 2= Uninterested, 3= Neutral, 4= Interested, 5= Very interested

Mark only one oval.

	1	2	3	4	5	
Very uninterested	⬭	⬭	⬭	⬭	⬭	Very interested

15. How satisfied are you with the quality of pre-clinical oncology teaching at your university? *

1= Very dissatisfied, 2= Somewhat dissatisfied, 3= Neutral, 4= Satisfied, 5= Very satisfied

Mark only one oval per row.

	1	2	3	4	5	N/A
Teaching hours	⬭	⬭	⬭	⬭	⬭	⬭
Content and material	⬭	⬭	⬭	⬭	⬭	⬭
Structure of the course	⬭	⬭	⬭	⬭	⬭	⬭

16. How satisfied are you with the quality of clinical oncology teaching at your university? *

1= Very dissatisfied, 2= Somewhat dissatisfied, 3= Neutral, 4= Satisfied, 5= Very satisfied

Mark only one oval per row.

	1	2	3	4	5	N/A
Teaching hours	⬭	⬭	⬭	⬭	⬭	⬭
Content and material	⬭	⬭	⬭	⬭	⬭	⬭
Structure of teaching	⬭	⬭	⬭	⬭	⬭	⬭

17. How many weeks of mandatory oncology teaching does your medical curriculum provide? Please provide an estimate of the total number weeks over the FULL duration of your medical degree. *

Mark only one oval.

- <1 week
- 1-2 weeks
- 3-4 weeks
- 4-6 weeks
- 6-8 weeks
- 8-10 weeks
- 10-12 weeks
- >12 weeks
- Not sure
- Other: ____________________

18. How do you think oncology teaching can be improved within your curriculum? *

____
____
____
____
____

End of questionnaire

Thank you for completing the NUOC 2020 pre-conference questionnaire.

All answers are de-identified and will be used only for the purposes described in the consent statement.

You will be receiving a post-conference questionnaire soon after the end of the conference; we highly encourage you to participate as well! (You will need your conference registration number or generated random ID for this questionnaire).

If you wish to withdraw your response and participation from this study, please email ha16849@qmul.ac.uk and quote your conference registration number or generated random ID.

This content is neither created nor endorsed by Google.

Google Forms
