# Supplementary material for: Inspiring the future generation of oncologists: a UK-wide study of medical students’ views towards oncology
Source: BMC Med Educ. 2021 Feb 2;21:82. doi: 10.1186/s12909-021-02506-0 (PMC7852146; doi:10.1186/s12909-021-02506-0)
Supplement: Supplementary file 3 — Additional file 3. Appendix 3. Post-conference questionnaire. [file 12909_2021_2506_MOESM3_ESM.pdf]

NUOC 2020 Post-Conference Questionnaire

* Required

Project description and consent statement

Dear Participant,

As a part of the National Undergraduate Oncology Conference 2020, we would like to invite you to fill in this post-conference questionnaire similarly to the pre-conference questionnaire you may have already answered. Please read the following to gain a better understanding of what this will involve, should you choose to participate.

As this is the first time an oncology conference of this scale is held, we would like to understand who you are as a participant, and what your existing perceptions of oncology are. This will include a series of questions about your thoughts and feelings on oncology and its different sub-specialties. You will also have the chance to evaluate the conference and provide feedback at the end. We will not collect your name or any other personal information that will make you identifiable. This questionnaire will take approximately 5-10 minutes. You will require your conference registration number or generated random ID to complete this questionnaire.

By completing the survey, you will help us understand what people are interested in, how to improve future conferences, and if the conference has made any impact or generated any new interest.

A few important points:
- We intend on publishing these results in an academic journal, however your answers will be kept confidential and your responses will be completely de-identified (i.e. all possible identifying characteristics, such as your conference ID, will be separated from your answers and any publicly available data).
- The data we collect will not be used for any other purpose other than those stated above.
- You have the right to withdraw at any time, before or after you submit the questionnaire, if you wish to do so for any reason. You will need to quote your conference registration number or generated random ID to withdraw your response after submission.

If you have any questions please contact ha16849@qmul.ac.uk.

1. I have read and understood the above information and I voluntarily consent to participate in this study. *

Mark only one oval.

- ◯ Yes
- ◯ No

2. Please provide your conference registration number *

This will be used to MATCH your responses to the pre- and post-conference questionnaires. You may withdraw your data after completing the questionnaire by quoting this number, should you wish to do so. If you do not have a conference registration number, please generate a random ID by putting the last 4 digits of your student ID number and first 2 letters on your surname (e.g. if your student number is 123456789 and your surname is Smith, your generated random ID is 6789SM. PLEASE USE THE SAME ID YOU ENTERED IN THE PREVIOUS QUESTIONNAIRE

Part 1: Views on oncology

You will have completed an identical version of this in the pre-conference questionnaire. This is for us to compare how your views may or may not have changed after the conference. Please be honest, all answers are de-identified!

3. How likely are you to pursue a career in oncology? *

1= Very unlikely, 2= Unlikely, 3= Unsure, 4= Likely, 5=Very likely

Mark only one oval.

	1	2	3	4	5	
Very unlikely	⬭	⬭	⬭	⬭	⬭	Very likely

4. Rate your interest in the following oncology career pathways. *

1= Very uninterested, 2= Uninterested, 3= Neutral, 4= Interested, 5= Very interested

Mark only one oval per row.

	1	2	3	4	5
Clinical research (e.g. clinical trials)	⬭	⬭	⬭	⬭	⬭
Scientific research (e.g. laboratory, pre-clinical)	⬭	⬭	⬭	⬭	⬭
Clinical oncology	⬭	⬭	⬭	⬭	⬭
Medical oncology	⬭	⬭	⬭	⬭	⬭
Surgical oncology	⬭	⬭	⬭	⬭	⬭
Palliative care	⬭	⬭	⬭	⬭	⬭

5. Rate how much you agree with the following statements. *

1= Highly disagree, 2= Somewhat disagree, 3= Neutral, 4= Somewhat agree, 5= Highly agree

Mark only one oval per row.

	1	2	3	4	5
Oncology is a challenging specialty.	○	○	○	○	○
If I were an oncologist, I am afraid that I would be overly sensitive.	○	○	○	○	○
If I were an oncologist, I am afraid that I would be too thick-skinned.	○	○	○	○	○
If I were an oncologist, I feel like I would be able to cope with the emotional challenges in this field.	○	○	○	○	○
I am overall optimistic about cancer as a whole.	○	○	○	○	○
I am overall pessimistic about cancer as a whole.	○	○	○	○	○

6. Rate how much knowledge or exposure you have had in the following aspects of oncology. *

1= Very little/ none, 2= Minimal, 3= Some, 4= A fair amount, 5= A lot

Mark only one oval per row.

	1	2	3	4	5
Career and specialty training pathway	○	○	○	○	○
Patient pathway (from diagnosis to treatment to recovery)	○	○	○	○	○
Patient experience and views	○	○	○	○	○
Types of cancer research and how they are carried out	○	○	○	○	○
Understanding of the different multi-disciplinary members and their roles in the cancer pathway	○	○	○	○	○

7. Rate your confidence in the following: *

1= Not confident at all, 2= Minimally confident, 3= Somewhat confident, 4= Quite confident, 5= Very confident

Mark only one oval per row.

	1	2	3	4	5
Communicating with a cancer patient	◯	◯	◯	◯	◯
Speaking about death and dying with a cancer patient	◯	◯	◯	◯	◯
Identifying skin cancer lesions	◯	◯	◯	◯	◯
Knowledge of the organisation and important aspects that govern clinical trial research	◯	◯	◯	◯	◯
How to build your CV towards a career in oncology	◯	◯	◯	◯	◯
Knowledge of the role of interventional radiology in diagnosing and treating cancer	◯	◯	◯	◯	◯

Part 2: Conference experience

This section is for us to understand what we did well and what could be done better about the conference. Please be honest, all answers are de-identified!

8. How satisfied are you with the following general aspects of the conference? *
1= Very dissatisfied, 2= Somewhat dissatisfied, 3= Neutral, 4= Satisfied, 5= Very satisfied

Mark only one oval per row.

	1	2	3	4	5
Overall oragnisation	◯	◯	◯	◯	◯
Relevance and engagement of lectures and workshops	◯	◯	◯	◯	◯
Appropriate level of delivery	◯	◯	◯	◯	◯

9. How would you rate the quality of the talks? *

1= Very poor, 2= Poor, 3= Neutral, 4= Good, 5= Very good

Mark only one oval per row.

	1	2	3	4	5	N/A
Translational trials in prostate cancer	◯	◯	◯	◯	◯	◯
Breast cancer and iKnife	◯	◯	◯	◯	◯	◯
Paediatric brain tumour research	◯	◯	◯	◯	◯	◯
Paediatric haemato-oncology case studies	◯	◯	◯	◯	◯	◯
Patient panel	◯	◯	◯	◯	◯	◯

10. How would you rate the quality of the workshops? *

1= Very poor, 2= Poor, 3= Neutral, 4= Good, 5= Very good

Mark only one oval per row.

	1	2	3	4	5	N/A
Skin cancer identification	◯	◯	◯	◯	◯	◯
Interventional radiology	◯	◯	◯	◯	◯	◯
Clinical research	◯	◯	◯	◯	◯	◯
CV building and academic foundation year program	◯	◯	◯	◯	◯	◯
Patient interaction	◯	◯	◯	◯	◯	◯

11. What were the overall highlights of the conference? What about the talks and workshops did you enjoy?

12. What are some things you did not enjoy or think can be improved overall?

13. Would you recommend this conference to someone else interested in oncology? *

1= Very unlikely, 2= Unlikely, 3= Unsure, 4= Likely, 5=Very likely

Mark only one oval.

	1	2	3	4	5	
Very unlikely	◯	◯	◯	◯	◯	Very likely

14. Would you attend a similar event in the future? *

1= Very unlikely, 2= Unlikely, 3= Unsure, 4= Likely, 5=Very likely

Mark only one oval.

	1	2	3	4	5	
Very unlikely	◯	◯	◯	◯	◯	Very likely

15. Do you think such conferences have an educational value in addition to pre-existing medical school teaching? *

Mark only one oval.

- Yes
- No
- Maybe

16. Any other comments about the conference?

End of questionnaire

Thank you for completing the NUOC 2020 post-conference questionnaire.

All answers are de-identified and will be used only for the purposes described in the consent statement.

If you wish to withdraw your response and participation from this study, please email ha16849@qmul.ac.uk and quote your conference registration number or generated random ID.

This content is neither created nor endorsed by Google.

Google Forms
