# Supplementary material for: Inspiring the future generation of oncologists: a UK-wide study of medical students’ views towards oncology
Source: BMC Med Educ. 2021 Feb 2;21:82. doi: 10.1186/s12909-021-02506-0 (PMC7852146; doi:10.1186/s12909-021-02506-0)
Supplement: Supplementary file 4 — Additional file 4. Appendix 4. Ethical approval. [file 12909_2021_2506_MOESM4_ESM.pdf]

Queen Mary, University of London
Room W117
Queen's Building
Queen Mary University of London
Mile End Road
London E1 4NS

Queen Mary Ethics of Research Committee
Mantalena Sotiriadou
Research Ethics Facilitator
Tel: +44 (0) 20 7882 8002
Email: research-ethics@qmul.ac.uk

c/o Dr Michail Sideris
Blizard Institute of Cell and Molecular Science
Queen Mary University of London
Whitechapel
London

3rd March 2020

To Whom It May Concern:

Re: QMREC2348 - Nationwide survey of medical students' views on oncology and the utility of a National Undergraduate Oncology Conference as an educational tool

I can confirm that Kathrine Sofia Rallis has completed a Research Ethics Questionnaire with regard to the above research.

The result of which was the conclusion that the proposed work does not present any ethical concerns; is extremely low risk; and thus does not require the scrutiny of the full Research Ethics Committee.

Yours faithfully

Dr Mantalena Sotiriadou – Research Ethics Facilitator
