# Supplementary material for: Inspiring the future generation of oncologists: a UK-wide study of medical students’ views towards oncology
Source: BMC Med Educ. 2021 Feb 2;21:82. doi: 10.1186/s12909-021-02506-0 (PMC7852146; doi:10.1186/s12909-021-02506-0)
Supplement: Supplementary file 5 — Additional file 5. Supplementary Tables. [file 12909_2021_2506_MOESM5_ESM.docx]

# Supplementary materials

**Supplementary Table 1. Weeks of mandatory oncology teaching in medical school curriculum reported by national survey responders and conference attendees.**

| Number of weeks | Number of students (%) | |
| --- | --- | --- |
|  | **National survey** | **Conference** |
| <1 | 12 (7.2) | 1 (2.9) |
| 1-2 | 36 (21.7) | 4 (11.8) |
| 3-4 | 26 (15.7) | 4 (11.8) |
| 4-6 | 19 (11.4) | 5 (14.7) |
| 6-8 | 6 (3.6) | 5 (14.7) |
| 8-10 | 5 (3.0) | 2 (5.9) |
| 10-12 | 3 (1.8) | 0 |
| >12 | 4 (2.4) | 0 |
| Not sure | 55 (33.1) | 13 (38.2) |

**Supplementary Table 2. Conference feedback: responses from post-conference questionnaire**.

| Question | Response^a^ |
| --- | --- |
| How satisfied are you with the following general aspects of the conference |  |
| Overall organisation | 4.3 ± 0.8 |
| Relevance and engagement | 4.4 ± 0.8 |
| Appropriate level of delivery | 4.4 ± 0.8 |
| How would you rate the quality of the talks? |  |
| Translational trials in prostate cancer | 4.5 ± 0.7 |
| Breast cancer and iKnife | 4.6 ± 0.5 |
| Paediatric brain tumour research | 4.2 ± 0.8 |
| Paediatric haemato-oncology case studies | 4.4 ± 0.9 |
| Patient panel | 4.4 ±0.6 |
| How would you rate the quality of the workshops? |  |
| Skin cancer identification | 4.2 ± 0.7 |
| Interventional radiology | 4.6 ± 0.6 |
| Clinical research | 4.3 ± 0.7 |
| CV building and academic foundation year program | 4.1 ± 1.0 |
| Patient interaction | 4.3 ± 0.9 |
| Would you recommend this conference to someone else interested in oncology? | 4.5 ± 0.7 |
| Would you attend a similar event in the future? | 4.5 ± 0.7 |

^a^ Data is reported as the mean value of the Likert score ± standard deviation.
